# Resistance Training Using Different Hypoxic Training Strategies: a Basis for Hypertrophy and Muscle Power Development

**DOI:** 10.1186/s40798-017-0078-z

**Published:** 2017-03-17

**Authors:** Belén Feriche, Amador García-Ramos, Antonio J. Morales-Artacho, Paulino Padial

**Affiliations:** Department of Physical Education and Sport, Faculty of Sport Sciences, University of Granada, Crta Alfacar sn, 18011 Granada, Spain

## Abstract

The possible muscular strength, hypertrophy, and muscle power benefits of resistance training under environmental conditions of hypoxia are currently being investigated.

Nowadays, resistance training in hypoxia constitutes a promising new training strategy for strength and muscle gains. The main mechanisms responsible for these effects seem to be related to increased metabolite accumulation due to hypoxia. However, no data are reported in the literature to describe and compare the efficacy of the different hypertrophic resistance training strategies in hypoxia.

Moreover, improvements in sprinting, jumping, or throwing performance have also been described at terrestrial altitude, encouraging research into the speed of explosive movements at altitude. It has been suggested that the reduction in the aerodynamic resistance and/or the increase in the anaerobic metabolism at higher altitudes can influence the metabolic cost, increase the take-off velocities, or improve the motor unit recruitment patterns, which may explain these improvements. Despite these findings, the applicability of altitude conditions in improving muscle power by resistance training remains to be clarified.

This review examines current knowledge regarding resistance training in different types of hypoxia, focusing on strategies designed to improve muscle hypertrophy as well as power for explosive movements.

## Key Points

- Despite the fact that the balance of results tends to favor resistance training in hypoxia, no consistent differences in results have been detected between hypertrophy/strength resistance training in normoxia and hypoxia. Moderate resistance loads and moderate altitudes may promote the most favorable physiological and functional changes.
- Ascent to altitude, as opposed to simulated hypoxia, leads to velocity and power improvements that could have positive applications in improving velocity and technical skills in power-related sports. The mechanisms that promote the benefit of this type of hypoxia over normobaric hypoxia still require clarification.
- Further research is needed to clarify the efficacy of resistance training specifically oriented to muscle power or hypertrophic gains. Additional research should involve trained athletes and take into consideration nutrition, hydration, and the adjustment of the training load at terrestrial altitude, before proposing new strategies for hypertrophy.

## Introduction

Altitude training is frequently part of an elite athlete’s exercise program. By inducing tissue hypoxia due to a lower arterial partial pressure of oxygen (PO_2_), altitude training causes a physiological response that affects performance. Traditionally, the ascent to a higher altitude has been associated with impaired endurance performance [1, 2]. However, when remaining at altitude, changes in the body systems involved in aerobic energy supply seem to elicit beneficial chronic adaptations improving performance [3, 4]. Conversely, non-oxidative metabolism-dependent, short-lasting activities (under 1 min) seem to offer immediate benefits when performed in altitude conditions [1, 5].

A hypoxic environment appears to create advantageous responses in the development of muscle performance with increased hypertrophy and gains in both muscle strength and speed of explosive movements. Despite the importance of resistance training to performance, the muscle response under conditions of hypoxia has not been studied in detail. Very few studies have evaluated the effect of induced hypoxia on anaerobic and aerobic metabolism or the capacity for recovery from different work/rest ratios in repeated exercise training [6, 7], as used during resistance training. The combination of the load, sets, repetition of sets, rest, and speed of movement are key factors in understanding the physical and functional muscle changes derived from specific resistance training, as well as the influence that “hypoxic conditions” could have on the results. For hypertrophy and strength gains, when looking for increased muscular cell swelling and metabolite accumulation, traditional methodology usually combines 6–12 sets of 8–12 repetitions at low velocity with loads of 65–80% of 1 repetition maximum (1RM) and 1–3 min of rest in between sets [8, 9]. For muscle power resistance training, geared to a neuromuscular goal and avoiding metabolic fatigue, sessions usually combine 4–6 sets of 4–6 repetitions with loads of 0–50% 1RM for ballistic exercises [10] and 3–5 min of rest (sometimes this method includes inter-repetition rests of 10–60 s, cluster training).

Given different both resistance training purposes, and considering the limited number of studies that have examined muscular adaptation and performance under hypoxic conditions, it is necessary to analyze the differences in the experimental designs, participant training levels, and the type and severity of hypoxia before drawing conclusions. In the following section, muscle hypertrophy and power trainability under hypoxic conditions will be reviewed in order to clarify the consistency of the results available.

Throughout the different sections of this review, different types of hypoxia and hypoxic training strategies will be referred to. Current training trends in hypoxia combine different types and dosages of hypoxia (H) resulting in numerous possible combinations [11, 12]:

- Hypobaric hypoxia (HH), or altitude, produces the hypoxic effect (decrease the availability of oxygen in the tissues) by reducing the barometric pressure, mainly by ascent to altitude or by using hypobaric chambers. The reduction in barometric pressure reduces air resistance to the movement as a result of lower air density. An ascent to altitude also results in reduced air temperature and humidity.
- Normobaric or systemic hypoxia (NH) provides the hypoxic effect by reducing the oxygen pressure in the inspired air (nitrogen dilution or oxygen filtration).

In this sense, natural or artificial methods of hypoxia can be used for training, resulting in strategies such as *live high-train high* (LHTH), *live high-train low* (LHTL), or *live low-train high* (LLTH) among others. Intermittent hypoxic devices are also used during training sessions (IHT) or resting periods. Training at low altitude allows the athlete to maximize performance by maintenance of sea-level training intensity and oxygen flux [11]. Extensive literature relating to the use of these combinations in endurance training exists, although its application in resistance training is mainly limited to the use of intermittent artificial hypoxic exposure while resting under normoxic conditions (LLTH) to increase hypertrophy, also called *intermittent hypoxic resistance training* (IHRT) [13].

## A Description of the Mechanisms and Metabolic Factors Related to the Hypertrophic and Functional Muscle Response in Acute and Chronic Hypoxic Conditions

**Fig. 1:**
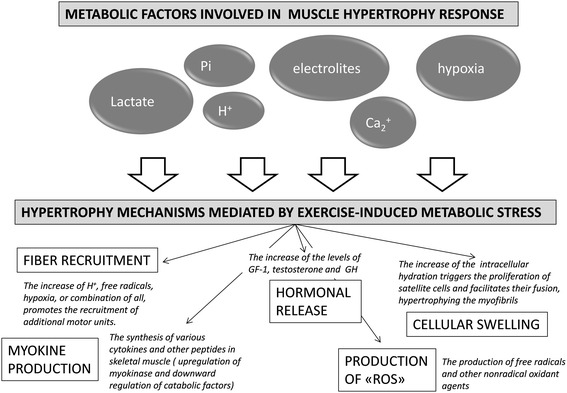
Hypertrophy mechanisms mediated by metabolites

Several studies have tested the degree to which hypoxic conditions are able to produce beneficial changes to muscle strength and hypertrophy [14–17]. These studies follow on from previous research into the apparent beneficial effects on muscle hypertrophy and strength gains of low resistance training (20–50% 1RM) when combined with blood flow restriction (BFR) in different subject populations [18–24]. The main mechanisms proposed for these improvements relate to responses to the metabolite build-up [25–27] (Fig. 1). Then, moderate intensity resistance training under hypoxic conditions enhances exercise-induced metabolic stress mechanisms (anabolic hormones, cytokines, reactive oxygen species, and oxidative stress factor, among others), which clearly have an important role in muscle growth [17, 27–30]). Accordingly, several studies have been conducted in hypoxia aiming to achieve strength and hypertrophic benefits [14, 17, 21, 22, 30, 31].

On the other hand, improvements in sprinting, throwing, and jumping at altitude have been reported [1, 2, 5, 32]. To date, researchers have mainly used the hypothesis of reduced energy costs to account for improved isolated high-speed actions [33] through a reduction in aerodynamic resistance in approximate proportion to the square of the velocity (e.g., when cycling, running, or throwing objects) [1, 5, 32]. However, although the underlying mechanisms are not completely defined, modified motor unit recruitment patterns due to increased anaerobic metabolism release [34, 35] could also partly explain these improvements. Furthermore, it could be considered that if this occurs in isolated movements, some benefit could also be obtained during a prolonged period of specific explosive resistance training at altitude, opening up a new line of investigation that considers the effects of both air composition and its resistance.

**Fig. 2:**
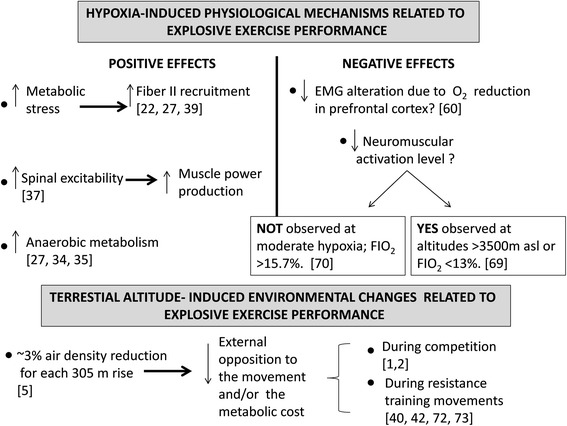
Hypoxia ascent and/or exposition-induced physiological mechanisms related to explosive exercise performance induced by hypoxia

Up until now, the impact of whatever type of hypoxia on muscle performance and the biomechanics of specific movements in sport has not been examined in depth. Alterations in the biomechanical and neuromuscular components associated with force production are some of the factors recently suggested by Chapman et al. [36] that influence changes in performance following altitude training. According to this, the improvement in speed movement can also be attributed to an enhanced firing frequency of motoneurons and spinal reflexes. Acute simulated hypoxia has been related to the increase of the spinal excitability [37]. Additionally, Tomazin et al. [38] recently observed a greater increase in the H-reflex amplitude of the soleus muscle at a terrestrial altitude of 2320 m when compared to that at sea level (~35%; *p* < 0.05), a response that could be linked to a direct effect of hypoxemia on the supraspinal structures. There may, however, be further contributory mechanisms, such as increased anaerobic metabolism [27, 34, 35] and/or reduced air density, as mentioned above [33], that influence muscle contraction properties and thus improve explosive speed [1, 5] (Fig. 2). In fact, breathing hypoxic gas mixtures while training seems to display a tendency for fast fiber areas to increase in size following training compared to breathing normoxic air [39] that could be also linked to a greater fast fiber recruitment. Accordingly, other studies have revealed maximal power gains during a force-velocity (*F*-*V*) curve [40–43], as well as 1RM gains [40–42], after acute exposure to real moderate altitude in bench press, back half squat, and squat jump exercises. However, Scott et al. [44] detected no changes in force and power performance during back squat and dead lift exercises (80% 1RM) at simulated moderate and high altitudes.

## Hypertrophy Trainability in Conditions of Hypoxia

Scott et al. [24] reviewed the hypothetical benefits on muscle strength and muscle size of intermittent hypoxic resistance training compared to BFR. Resistance training under conditions of systemic hypoxia (NH; achieved by nitrogen dilution, oxygen extraction in an environmental chamber or wearing a face mask) could be considered as an alternative to BFR while avoiding problems such as restricting training to only the limbs, pain, petechial hemorrhage, and dizziness related to the use of compression cuffs [24]. As has been mentioned, moderate intensity resistance training under hypoxic conditions enhances the build-up of metabolites, which clearly have an important role in muscle growth [17, 27–30]. However, the literature does not provide data addressing the effect of a resistance training period at terrestrial altitude, and conclusions obtained during IHRT in normobaric hypoxia are not clear. Gains in muscle mass and strength after a hypoxic training period display no consistent differences when compared with those observed in normoxia (Table 1).

**Table 1:** Studies assessing muscle strength and hypertrophy responses to resistance training under conditions of hypoxia

	*n*	N-CG (*n*)	Training level	H cond.	Level of hypoxia	Training intervention			Additional hypoxia effects with respect to the CG	
						Weeks (s/w)	Volume	Exercise	Muscle strength	Muscle structure
Friedman et al. [14]	19	Yes (9)	Untrained	NH (H room)	12% FiO_2_	4 weeks (3)	30% 1RM 6 × 25 reps. (Rest 60 s )	Knee extension	1RM (ns) MIKS (ns) End-50 (ns)	CSA (ns) Fiber TD (ns)
Ho et al. [15]	18	Yes (9)	Untrained	NH (hypoxic tent)	15% FiO_2_ (+10 min pre- and post-training)	6 weeks (3)	10RM 3 × 10RM (Rest 120 s)	Back squat	1RM (ns) MIS-60 (ns) MIKS 60/s (ns)	
Kon et al. [16]	16	Yes (7)	Untrained	NH (hypoxia room)	14% FiO_2_ (+10-15 min pre- and 30-60 min post-training)	8 weeks (2)	70% 1RM 5 × 10 (Rest 90 s)	Bench press Leg press	1RM (ns)	CSA (ns)
Kurobe et al. [17]	13	Yes (7)	Untrained	NH (hypoxia room)	13% FiO_2_ (+30 min pre-training +60 min post-training)	8 weeks (3)	10RM 3× rep. to failure (Rest 60 s)	Elbow extension	10RM (ns)	
Manimmanakorn et al. [21, 22]	30	Yes (10)	Well-trained netball players	NH (face mask)	80% SaO_2_	5 weeks (3)	20% 1RM 3× rep. to failure (Rest 30 s)	Knee flexion Knee extension	MIS-80°▲ MIS-30▲ End-20▲	CSA ▲
Nishimura et al. [31]	14	Yes (7)	Untrained	NH (hypoxia room)	16% FiO_2_ (+30 min pre- and post-training)	6 weeks (2)	70% 1RM 4 × 10 reps. (rest 60 s)	Elbow extension Elbow flexion	1RM (ns)	CSA (ns)

*n* sample size, *N-CG* normoxia control group (sample size), *H cond*. hypoxia condition, *NH* normobaric hypoxia, *FiO*_*2*_ inspired fraction of oxygen, *Weeks s/w* number of session performed in the week, *volume %RM* load expressed as a percentage of 1 repetition maximum, *sets × repetitions* interset rest period, *MIS* maximal isometric strength, *MIKS* maximal isokinetic strength, *End-n* strength endurance capacity test of *n* repetitions, *CSA* cross-sectional area, *fiber TD* fiber type distribution, ▲ increase, ▼ decrease, *ns* non-significant change

The effect of the hypertrophic resistance training at terrestrial altitude on muscle mass has not been studied in detail. Early studies associated the ascent and extended periods spent at high altitude (>5500 m) with up to a 15% muscle mass loss [45] and reduced strength gains (−6.4%) compared to normoxia [46]. Explanations for this have included altitude-induced protein synthesis rate reduction [47, 48] or increased protein degradation during exercise [49], leading to a negative synthesis/degradation of protein balance. Nevertheless, training camps are usually held at moderate altitudes (1800–2500 m), while no data are reported in scientific literature about hypertrophic resistance training during intermittent or chronic periods at terrestrial altitude; protein metabolism seems to be unaffected by O_2_ availability at moderate simulated altitudes in acute NH [50].

### Effect of Low-Load Resistance Training on Hypertrophy in Conditions of Hypoxia

Several interesting studies have been analyzed in order to highlight the effect of low-load resistance training in hypoxia on strength and muscle growth (see Table 1). Additional muscle size gains of 3.2% were detected by Manimmanakorn et al. [21, 22] after 5 weeks of low-intensity resistance training under intermittent normobaric hypoxia (SaO_2_ of 80%, ~4000 m asl) when compared with normoxia (3 sets of the repetitions to failure at 20% 1RM, 30 s of rest between sets). Traditionally, multiple sets of loads over 65–70% of 1RM were considered necessary for significant hypertrophy [8]. However, current trends suggest that low-load resistance training is also able to induce muscular hypertrophy through mechanisms not related to mechanical stress. In this case, muscle growth is highly dependent on metabolic factors, and training sets should be conducted to failure [27]. These two points must be considered in both normoxic and hypoxic conditions despite the fact that there is an accelerated build-up of metabolites observed in hypoxia during resistance training with moderate loads [17, 27–30], but not with low loads [51]. For instance, similar lactate and anabolic hormone responses were observed for squat exercises (5 sets of 15 repetitions at 30% 1RM, 90 s of rest) in conditions of normoxia, as those for systemic hypoxia (15% of the inspired oxygen fraction [FiO_2_]) [51]. For this reason, performing a high number of repetitions in each training set is an important detail that could explain the positive results of Manimmanakorn et al. [21, 22] given the absence of strength and hypertrophic changes in other studies under similar conditions [14]. Manimmanakorn et al. [21, 22] compared three experimental situations: normoxia, normobaric hypoxia, and BFR, and participants were instructed to match the repetitions performed by the BFR group to ensure equal training loads between groups (~28 and 36 repetitions in knee flexion and extension respectively). However, Friedmann et al. [14], when comparing the effect of 4 weeks of resistance training in normoxia and normobaric hypoxia (FiO_2_ = 12%; ~4500 m asl), used a fixed number of sets of repetitions in the same exercises (6 sets of 25 repetitions at 30% 1RM, 60 s of rest between sets). Additionally, the 30-s difference in rest periods between these studies could also have had a favorable influence on the Manimmanakorn et al. exercise routine as shorter recovery periods may heighten the metabolic stimulus to enhance the anabolic response [13]. Conversely, in these three studies [14, 21, 22], endurance-force was improved in hypoxia despite the exposure to lower SaO_2_ than in hypoxic dose recommendations (<3500 m or SaO_2_ >80%) [50], probably due to improved metabolic efficiency [22].

### The Effect of Moderate-Load Resistance Training on Hypertrophy in Conditions of Hypoxia

When analyzing research conducted by using moderate resistance training programs (65–80% 1RM), three of the four available studies (see Table 1) display results which do not reveal a clear effect of systemic hypoxia on muscle strength and hypertrophy with respect to the same training in normoxic conditions. Accordingly, the influence of acute exercise-induced endocrine responses in muscle growth has recently been questioned, and may not have the expected anabolic effects in healthy subjects [52]. This contrasts with the evidence described in other studies in which a hypertrophic resistance training program produced strength gains [31], and muscle cross-sectional area (CSA) increases driven by the growth hormone response [26].

Compared to normoxic conditions, Nishimura et al. [31] observed that 6 weeks of resistance training (4 sets × 10 repetitions at 70% 1RM; 1 min rest) under normobaric hypoxic conditions (16% FiO_2_) improved arm strength levels (66 vs 48%, non-significant), and a change in CSA in hypoxia was shown at early stage throughout the training period (1.3–1.9% increase in CSA) in untrained subjects. Similar results were reported by Kurobe et al. [17], who associated the better results of 8 weeks of resistance training of the arms (3 sets of 10 1RM; 1 min of rest) while breathing a reduced O_2_ air (13% FiO_2_) with higher growth hormone secretion. This is consistent with the greater blood lactate and anabolic hormone responses observed by Kon et al. [28] after a bench press and leg press resistance training session (5 sets of 14 repetitions at 50% 1RM; 1 min rest) in NH conditions of 13% of FiO_2_ despite the fact that the load used was just below the low-limit threshold of what is considered a “moderate-load” (but nevertheless above what is considered a lower load). No differences between conditions in the theoretical maximal strength were reported by Kurobe’s team, but 1RM estimates in high repetition tests to failure (10RM, ~75% 1RM) may be unreliable due to fatigue and mechanical stress [53, 54]. Contrary to these findings, Ho et al. [15] concluded that 6 weeks of short-term resistance training (3 sets of 10RM; 2 min rest) under NH (15% FiO_2_) offered no additional benefit to muscular performance or body composition. Likewise, Kon et al. [16] observed no strength or muscle size gains in response to bench press and leg press (16 sessions of 5 sets × 10 repetitions at 70% 1RM; 90 s of rest) though they did detect enhanced skeletal muscle endurance and angiogenesis.

### Inconsistencies Among Studies of Hypertrophic Resistance Training Effects Mediated by Hypoxia

The lack of consensus among reported studies may be attributed to differences in protocols such as training intensity (light or moderate loads), number of sets (3 to 6), rest period between sets (30 to 120 s), muscles worked (arms, legs, chest), training program duration (4 to 8 weeks), and severity of hypoxia (from 12 to 16% FiO_2_). Additionally, all studies were performed in untrained subjects and the muscle stimulus employed was lower than recommended for hypertrophy [8, 55]. In untrained subjects, neural modifications start during the early stages of training [14, 56] with an individual long-phase that could, in part, explain the different strength and/or muscle size gains observed among studies.

In the reviewed studies, hypoxia levels were performed at simulated hypoxia which ranged from 12 to 16% FiO_2_, which although being at or slightly above the limit (~12% FiO_2_) is not likely to produce discrepancies. During 3.5 h following moderate-intensity resistance training (6 sets of 8 repetitions at 70% 1RM) under acute severe hypoxia (~4300 m asl, 12% FiO_2_), a relationship exists between protein synthesis rate and arterial oxygen saturation (SaO_2_) (*r* ^2^ = 0.49, *p* = 0.04). This means that for SaO_2_ <80% or altitudes >3500 m, hypoxia may delay the anabolic response to resistance training compared to normoxia, although after 3.5 h, responses should be comparable [50]. No data concerning the effect of hypertrophic resistance training at terrestrial altitude are available, so differences between simulated and terrestrial hypoxia cannot be reported. At high or moderate altitude, other factors could also promote muscular changes over longer periods, such as the following: (1) reduced food intake (10–50%) linked to a loss of appetite [57] and/or change in diet; (2) increased energy expenditure due to a higher basal metabolic rate [58] and/or physical activity not matching energy intake; (3) dehydration [59]; and (4) absence of load adjustment during hypertrophic resistance training at altitude. Indeed, the reduction of the training load stimulus to the muscle during altitude training can be considered as one of the mediating factors related to the loss of muscle mass traditionally linked to an altitude camp. This can occur when the training load used in normoxia is maintained during the altitude stage. Therefore, if the 1RM absolute load improves by HH and the resistance training load is not adjusted accordingly, the stimulus to the muscle during the resistance altitude training will be reduced. In this context, Feriche et al. [40] describe a concentric bench press 1RM improvement at acute terrestrial altitude (~5.6%; ES = 1.1) with respect to the change observed in normoxia and NH. Similar results have also been described in the half squat [41]. However, there are no data from any longitudinal study at terrestrial altitude.

Finally, another point of consideration is that the minimum time needed to detect significant hypertrophic muscular changes in athletes is around 8 weeks [9]. The research revised deals with study periods ranging from 4 to 8 weeks, which could contribute to the discrepancies observed among the results, as well as limiting the use of terrestrial altitude. Changes in muscle size and strength resulting from resistance training during an extended period at real moderate altitude still require clarification. Moreover, real moderate altitude training camps normally last 3 weeks, which is an insufficient time to achieve the target, although the hypertrophic training must be carefully adapted to avoid the undesired results previously mentioned. For this reason, when pursuing hypertrophy, longer hypoxic training programs which simulated normobaric hypoxia is generally selected, usually involving IHRT sessions.

According to Scott et al. [13], more control research is needed to evaluate the real influence that hypoxia constitutes in encouraging hypertrophy and strength gains. The potential effect of hypoxia on growing muscle mass, specially by the marked metabolic stress that the exercise performed under hypoxic conditions causes, is well documented [27, 28, 30] although questioned in some contexts by the lack of positive results [14–16]. This information should be considered in future investigations conducted in hypoxia and take into account previous conclusions about how to apply hypertrophic methods [8, 9], type and severity of the hypoxia [11, 12, 50], influence of the training level on the sample [14, 56], and recommended length of the intervention [9]. Moreover, the ideal strategies for resistance training during altitude camps and other types of interventions combining terrestrial altitudes should also be explored.

## Muscle Power Trainability in Conditions of Hypoxia

The influence of altitude or hypoxic conditions on muscular function during muscle power resistance exercises has not been examined in detail. Exercise-induced fatigue may have a central element which may or may not include a peripheral cause. Although the direct impact of hypoxia on the brain cannot be ruled out [60], sensory feedback of metabolite accumulation due to lower O_2_ availability (such as H^+^ or Pi) may explain why the central command and power output in hypoxia is reduced [61]. Literature describes a direct but moderate influence of the inspired O_2_ fraction on the central nervous system. This conclusion was reached by Millet et al. [62] after studying the response of intermittent isometric unilateral knee extensions to failure with and without blood flow restriction (BFR, via a cuff), in N and while breathing a reduced O_2_ air (NH of 11% FiO_2_ and 84% SaO_2_). Both, hypoxia and the occlusion cuff, affected the number of repetitions. However, considering the muscle similarly affected in the two conditions, performance was slightly but significantly lower during NH than in N with cuff on. The design used in this study leads us to conclude that systemic hypoxia has a direct influence on the central drive, independent of the factors developed within the working muscles. During severe levels of hypoxia, the type of muscle contraction or the total muscle mass involved in exercise can also limit the influence of this mechanism [62].

Perrey and Rupp [63] reviewed studies analyzing the effects of acute or prolonged exposure to terrestrial high altitude (≥3700 m) on the contractile properties of the muscle. Under conditions of acute altitude, muscle function was altered after intermittent contractions, while modifications to chronic hypoxia seemed to minimize the effect on skeletal muscle function. Impaired muscle function in response to high altitude was especially appreciable during exercise protocols involving prolonged isometric muscle contractions (<30% of the maximal voluntary contraction), and during repeated submaximal intermittent contractions, both of which depend principally on systemic O_2_ transport [63]. Besides muscle deterioration due to high altitude, reduced muscular power has also been described [64].

In contrast, terrestrial moderate altitude throws up different results. Two previous studies reviewed the effect of altitude on elite athletes’ performance in sprints from 100 to 400 m [1, 2], and throwing and jumping performance [1]. As was predicted by mathematical models [33], enhanced sprint performances (0.2–0.7%), the hammer throw and triple and long jump (~1%) are described at altitudes above 1500 m [1]. Nowadays, there is no doubt about the benefit of terrestrial altitude on these athletic disciplines. Nevertheless, some authors suspect that the slower take-off speeds in high jump, or the slower velocity in the hurdle race (i.e., from 10.3 to 8.5 m s^−1^ in 100 m sprint and 110 hurdles, respectively, in men), reduces the influence of aerodynamic drag and it is this that accounts for the minimal effect on performance between altitudes [1]. However, despite the greater difference in speed compared with sprints, jumps, and throws, the changes described in muscle power and velocity during isolated resistance exercises (back squat, bench press or squat jump) from the first hours following ascent [40–43] indicate that some kind of relationship between HH and muscular function must exist, independently of the changes in speed linked to air density (Tables 2 and 3).

**Table 2:** Studies assessing the acute effects of altitude on explosive action performance

	*n*	Procedure (*n*)	Training level	H cond.	Level of hypoxia	Intervention		Hypoxia effects	
						Methodology/assessment	Exercise	Muscle strength	*F*/*V*/*P*
Feriche et al. [40]	28	2 groups Random trials G1(17): N and HH G2: N and NH (11)	Well trained in judo, taekwondo, and wrestling	HH NH Face mask	2320 m 16% FiO_2_ (+10 min pre-training)	*F*-*V* curve *F*-*V* curve	Bench press	1RM▲ 1RM (ns)	Load-*P*_max_▲ *P*_max_ (ns) *P*▲ (>60% norm. 1RM) *P*, *V*, *P*_peak_▲ (>60 kg) Load-*P*_max_ (ns) *P*_max_ (ns) *P* (ns at any % 1RM) *P*, *V*, *P*_peak_ (ns at any load)
Chirosa et al. [41]	5	Random trials N and HH	Physical education students	HH	2320 m	*F*-*V* curve 10 sets × 10 reps. (Rest 3 min) 70% 1RM	Back squat	1RM (ns)	*P*_max_ (▲) Load-*P*_max_▲ *P*, *V* (ns)
García-Ramos et al. [42]	18	Ramdom trials: N and HH	Elite swimmers	HH	2320 m	*F*-*V* curve	SJ		*P*_peak_▲ *V*_peak_▲
García-Ramos et al. [43]	17	N and HH	Elite swimmers	HH	2320 m	*F*-*V* curve Unloaded jumps	SJ CMJ and SJ		*P*_0_▲ *V*_0_▲ *F*_0_ (ns) *P*_peak_▲ *V*_peak_▲ *F*_peak_ (ns)
Scott et al. [44]	12	Random trials N and NH	Resistance trained	NH Face mask	16% FiO_2_ (+10 min before) 13% FiO_2_ (+10 min before)	80% 1RM 5 sets × 5 reps. (Rest 3 min)	Back squat Deadlift		*F* (ns) *P* (ns) *F*_peak_(ns) *P*_peak_(ns)

*n* sample size, *Procedure* (sample size), *N* normoxia, *H cond.* hypoxia condition, *NH* normobaric hypoxia, *HH* hypobaric hypoxia, *FiO*_*2*_ inspired fraction of oxygen, *Methodology* %RM (load expressed as percentage of 1 repetition maximum), *sets × repetitions* interset rest, *F*-*V curve* force-velocity curve, *F* mean force, *P* mean power, *V* mean velocity, *F*_*peak*_ peak force, *P*_*peak*_ peak power, *V*_*peak*_ peak velocity, *P*_*max*_ maximal power, *Load-P*_*max*_ load linked to maximal power, *F*_*0*_ theoretical maximal force, *V*_*0*_ theoretical maximal velocity, *P*_*0*_ theoretical maximal power, *SJ* squat jump, ▲ increase, ▼ decrease, *ns* non-significant change

**Table 3:** Studies assessing muscle explosive action response to resistance training under conditions of hypoxia

	*n*	Procedure (*n*)	Training level	H cond.	Level of hypoxia	Training intervention			Hypoxia effects
						Number/s	Methodology/assessment	Exercise	
García-Ramos et al. [42]	18	Ramdom trials N and HH	Elite swimmers	HH (2 weeks)	2320 m	10	3–4 × 6–12 reps. 30–90% BW *F*-*V* curve	Back-squat SJ	*P*_peak_▲ *V*_peak_▲
Álvarez-Herms et al. [71]	12	2 groups G1(7): N G2(5): sHH	Physical education students	sHH (4 weeks)	2500 m (+10 min pre-training)	12	4 × 15–25 reps. (90 s–2 min rest) 5 × 10 reps. (45 s rest) 5 × 5 reps. (3 min rest) Isolated jumps	Back-squat Jumps Jumps SJ CMJ	Jump Height (ns)
García-Ramos et al. [72]	15	Ramdom trials N and HH	Elite swimmers	HH (2 weeks)	2320 m	10	3–4 × 6–12 reps. 30–90% BW *F*-*V* curve *T*-15 m	Back-squat SJ	Jump Height ▲ T-15 m *▼*
García-Ramos et al. [73]	13	Trials in N and HH	Elite swimmers	HH (3 weeks)	2320 m	Pool, 2 s/day × 6 days/week Dry land, 1 s/day × 6 days/week	Concurrent strength and endurance training *F*-*V* curve *T*-5 m *T*-10 m *T*-15 m Start: take-off *V*	Squat, deadlift, leg ext, hip thrust SJ Swimming start	*T*-5 m (ns) *T*-10 (ns) *T*-15 (ns) *V*_peak_ (ns) Take-off *V* (ns)

*n* sample size, *Procedure* (sample size), *N* normoxia, *H cond.* hypoxia condition, *NH* normobaric hypoxia, *HH* hypobaric hypoxia, *sHH* simulated hypobaric hypoxia, *FiO*_*2*_ inspired fraction of oxygen, *Numbers/s* number of sessions, *Methodology ex/s* exercises per training session, *%RM* load expressed as percentage of 1 repetition maximum, *sets × repetitions* interset rest, *F-V curve* force-velocity curve, *P*_*peak*_ peak power, *V*_*peak*_ peak velocity, *BW* body weight, *SJ* squat jump, *CMJ* countermovement jump, *T-n* time to *n*-meters during a swimming start,▲ increase value,▼ decrease value, *ns* non-significant change

Very frequently, power and velocity assessed during the *F*-*V* curves are measured by means of linear transducers attached to the bar, making it difficult to identify if athletes applied more force at altitude or if the results are produced by lower resistance to movement. To analyze this, García-Ramos et al. [43] examined the leg extensor muscle response using a *F*-*V* curve and unloaded jumps (CMJ and SJ) in 17 elite swimmers before and just after an ascent to terrestrial moderate altitude. The novel aspect of this study is that during the *F*-*V* curve, the maximum values of force and velocity at each load were recorded by a force platform and a linear velocity transducer respectively. Thus, these variables were modeled by a linear regression [*F*(*V*) = *F*_0_ *aV*], where *F*_0_ (force intercept at zero *V*), *V*_0_ (velocity intercept at zero force), and maximum power output (*P*_0_ = *F*_0_ *V*_0/4_) were considered as the maximal mechanical capabilities of the neuromuscular system to generate force, velocity, and power, respectively [65]. The results revealed higher magnitudes in *P*_0_ (+6.79%; *p* < 0.01) of the leg extensors at altitude, which were linked to an increase in the *V*_0_ (+7.60%; *p* < 0.05), while no changes for the *F*_0_ (+0.02%) were achieved. In addition, the results for unloaded jumps performed on a force platform showed a clear tendency towards improvements in the amount of force applied when performed at altitude, with jump height increasing by an average of 3.4%. These results highlight the influence that the aerodynamic drag forces could have on velocity and show a clear altitude effect on the *F*-*V* relationship at the same absolute load [40, 42, 43], sustaining the hypothesis that the hypobaric hypoxia and muscular function relationship must, at some point, converge in addition with the additional benefit produced by the lower aerodynamic resistance on isolated explosive movements.

### Terrestrial or Simulated Altitude: Effect on Explosive Muscle Performance

Despite the reduced O_2_ content of air during a terrestrial or simulated exposure to hypoxia, differences in barometric pressure can also affect performance in repeated (IHRT) or isolated high-speed explosive actions. In 28 combat sport athletes divided into two homogeneous groups, *F*-*V* curves in the bench press were compared between N, terrestrial moderate altitude (HH 2320 m asl), and normobaric hypoxia (NH 15.7% FiO_2_) [40]. Results indicated a marked effect on the *F*-*V* curve of acute HH compared to negligible effects of N and NH. Acute HH led to a 3.2% mean increase in the load linked to mean maximal power, along with clear improvements in mean power, peak power, and peak velocity for the same absolute load. Hypobaric hypoxia also accounted for a 6% increase in 1RM after the ascent. This could be considered unsurprising given the confirmed relationship between mean velocity and weight lifted according to percentage 1RM [66–68], and the fact that this velocity is improved at terrestrial altitude [40, 42]. Using as a reference the 1RM recorded for N, the mean power curve for HH was shifted upwards and to the right, indicating that mean power would be overestimated for loads ≥60% of 1RM, compared to the curve obtained using as reference the corresponding 1RM recorded for HH [40]. The lack of power output change in NH concurs with the findings of Scott et al. [44]. Power and force trends over 5 sets of 5 repetitions at 80% of 1RM for acute moderate and high NH (13 and 16% FiO_2_) failed to vary from trends recorded in normoxic conditions.

According to other findings, and with evidence of physiological and metabolic responses induced by acute NH exercise (i.e., cardiovascular and hormonal [17, 28, 30, 44]), velocity, power, and maximum dynamic strength after basic strength exercises show benefits from HH which are not found in N or NH [40–42]. A relationship has also been identified between metabolic stress induced by H^+^ elevation because of low SaO_2_ and the recruitment of fast twitch muscle fibers [27]. While abnormalities in muscle electromyographic activity have been observed in conditions from acute hypoxia (~3500 m asl; 13% FiO_2_; [69]), moderate altitudes do not lead to these detrimental effects [70]. Additionally, electromyographic activity at NH has been shown to be similar to that at normoxia during maximal voluntary contractions and power output [22]. For isolated short-burst actions involved in *F*-*V* curves (~5 s plus 3–5 min rest), hypoxic benefits to performance were not observed in moderate or high NH (16–13% FiO2) [40, 44], and only improved at terrestrial altitude [40]. This challenges the idea that the breathing of air impoverished in O_2_ is solely responsible for inducing a switch from type I to II fibers [22] making the movement faster due the intrinsic capacity that larger motor neurons have to drive the impulses at higher speeds. It is likely that differences between results obtained at NH and HH seem mediated by other factors and/or interactions not yet investigated.

Álvarez-Herms et al. [71] did not observe any change in the height achieved during an isolated free squat jump (SJ) and countermovement jump (CMJ) after 4 weeks of endurance resistance training in simulated hypobaric hypoxia of 2500 m asl (*n* = 6 men and 1 women) and normoxic conditions (*n* = 3 men and 2 woman). This result is not surprising since the training was oriented to endurance. In contrast, a recent study conducted on 18 young male swimmers of a junior national team found mean peak power and peak velocity improvements of 12.1 ± 1.8% and 6.6 ± 1.2%, respectively, for loaded SJ after ascending to a terrestrial moderate altitude [42]. This study also demonstrated the persistence of altitude-induced improvements in jump performance after 2 weeks of exposure to real moderate hypoxia, showing mean improvements in both variables of 7.8 and 4.4%, respectively. Moreover, significant correlations between the percent change in jump height and the percent change in swimming start performance were also obtained following a short-term training program of 17 days [72]. These three studies show that moderate exposure to real or simulated hypobaric hypoxia does not impair the ability to apply force rapidly [42, 71, 72], and this capacity is likely to improve with specifically target-oriented training [42, 72]. In accordance with this hypothesis, it has been recently observed that 3 weeks of training high-living high at 2320 m asl does not produce adverse effects on muscular function in elite swimmers, even if the training is not solely focus on improving force and power [73]. In this study, the same group of swimmers were compared before and after 3 weeks of training at sea level at moderate altitude. Evaluations were separated by a 1-year period, although the intervention period, training targets, and relative loads were maintained. No changes in swimming start times were observed after the altitude period, while an impairment was registered at sea level. No differences between conditions were obtained during the loaded SJ performance despite a slight improvement in peak velocity after both training periods (Table 3).

The approximate 22.9% difference in air density at moderate altitude (~3% reduction for each 305 m rise; [5]) could contribute to making the movement faster than at normal altitudes. More studies are needed in order to analyze if an interaction exists between air pressure and composition, as well as examining the effects of longer training periods in these conditions. Researchers have identified [14, 74–76] and ruled out [77–79] differences in the physiological responses to exercise when comparing NH and HH. Millet et al. [76] reported power output improvements of 4.0 and 4.2% for elite and non-elite athletes, respectively, in conditions of HH vs 0.6 and 1.4% for NH. Bonetti and Hopkins [80] described increased ventilatory responses, changes in fluid balance, and nitric oxide metabolism, along with changes in the severity of acute mountain sickness and altered performance for HH compared to those for NH. However, these studies assessed the effects of chronic hypoxia, and no explanation has yet been offered for muscle power differences related to acute and longer periods spent at altitude.

### Inconsistencies Among Studies Conducted into the Effect of Hypoxia on Muscle Power Resistance Training

The influences of whatever type of hypoxia on muscle-specific performance in sport have not been thoroughly examined. While its causes remain unclear, we have identified a mechanism which could positively affect the performance of and training for isolated explosive actions at terrestrial moderate altitude. Muscle force or power development in hypoxic conditions is not a variable that is commonly assessed in scientific publications. From the nine studies included in this part of the review, five analyze the effect of acute exposure and contain measurements at different severities (FiO_2_ from 16 to 13%) and for different types of hypoxia (systemic vs terrestrial altitude). They examine the effect of various types of exercises (jumps, squat, deadlift, or bench press) and include subjects of differing sporting ability (elite vs non-trained). The remaining studies are conducted at real or simulated hypobaric hypoxia of around 2400 asl and analyze the effect of different types of resistance training on free or loaded jumps (SJ and CMJ). The presence of a control group, group size, sex distribution in groups, training level, or description of the training and assessment process are some of the differences observed among the abovementioned longitudinal studies. Finally, training orientation is also different among the studies. While Álvarez-Herms et al. [71] described resistance endurance training to improve anaerobic power during multiple jumps, García-Ramos et al. [42, 72] did not implement the study with specific tasks. The authors did however indicate that six of the ten resistance training exercises were oriented to strength-power training in the legs [42, 72]. Additionally, the concurrent strength and endurance training used in the García-Ramos et al. [73] study corroborate that an excessively oriented training aiming to improve endurance capacity attenuates strength training responses [81], even after an altitude training camp of 3 weeks [73, 82].

Controlled and power-oriented resistance training studies are clearly needed to analyze the effect of intermittent or sustained altitude exposure on power training.

## Conclusions

Current evidences suggest potentially promising applications of hypoxia for muscle hypertrophy and power training. Nevertheless, there is still insufficient data on which to base training programs. To help design altitude training protocols, data from more specific controlled studies are needed.

### In Hypertrophy

The evidence for greater muscle strength gains and structural physiological changes in response to resistance training under conditions of hypoxia is not conclusive. This is because although the balance of results tends to favor training in hypoxia, only one study revealed significant differences in performance between resistance training in normoxia and training in hypoxia.

Currently, the definitive mechanisms that may augment muscular responses to hypertrophy resistance training under hypoxic conditions are not yet fully understood. However, despite a need for further research, it may be reasonably suggested that (1) metabolite build-up during low-intensity (≤30% 1RM) resistance exercise may be intensified in hypoxia; (2) greater and faster changes may occur in hypoxia when multiple sets of 6–12 repetitions at moderate load (≥65% 1RM) are performed; and (3) the recommended simulated hypoxia level for all training modalities is moderate (13–16% FiO_2_).

Preliminary studies seem to indicate that hypertrophy-oriented training conducted under conditions of intermittent hypoxia could promote more favorable physiological and functional changes than under chronic exposure. Terrestrial or simulated *living low-training high* strategies seem to benefit anabolic responses.

Issues related to nutrition and hydration, as well as the adjustment of the training load due to the possible influence of an ascent in altitude on the 1RM estimation (to avoid reducing muscle stimulus and muscle mass), should also be taken into account when spending long periods at altitude.

### In Muscle Power Development

Ascent to altitude leads to velocity and power improvements although the mechanisms that promote the benefit of this type of hypoxia with respect to the NH still require clarification. Athletes should not be excessively concerned about the deterioration of muscular function when they take part of a 2–3-week training period at moderate altitude, even if the training is not strongly oriented to force and power development.

The following points should be considered in an altitude power-oriented training program: (1) loads used for power training under normal conditions should not be literally translated to training programs performed at higher altitudes. This is especially relevant because of the importance of locating and assessing the optimal muscular load for power training programs; (2) load adjustments during resistance training sessions at terrestrial altitude (according to the altitude 1RM) avoid reducing the muscle stimulus and/or inter- and intra-muscle coordination that commonly occurs after periods of altitude training; (3) *F*-*V* curves emerging from the different studies, despite involving different resistances, correspond to exercise volumes that do not induce local metabolic fatigue and could thus compromise muscle contractile properties. Protocols with inter-repetition or intra-set rest periods (cluster training) might therefore be more suitable for hypoxic resistance training focus on this topic.

Unlike simulated hypoxia, terrestrial altitude conditions seem to improve the ability to perform high-speed actions with moderate loads. Thus, training under these conditions could serve to improve velocity and technical skills in power-related sports.
